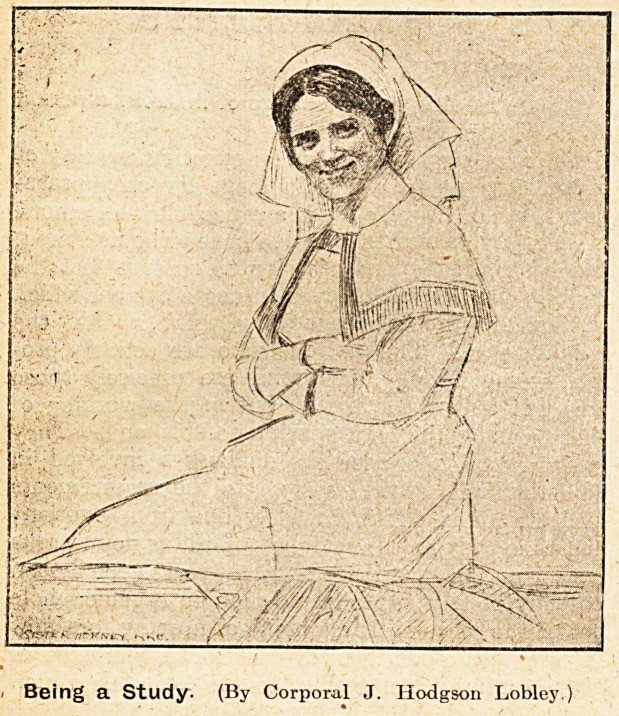# A Moment of Leisure

**Published:** 1917-12-22

**Authors:** 


					258  THE HOSPITAL December 22, 1917.
A Moment of Leisure.
Being a Study. (By Corporal J. Hodgson Lobley.

				

## Figures and Tables

**Figure f1:**